# Preparation and Characteristics of Ethylene Bis(Stearamide)-Based Graphene-Modified Asphalt

**DOI:** 10.3390/ma12050757

**Published:** 2019-03-05

**Authors:** Xia Zhang, Jun-Xi He, Gang Huang, Chao Zhou, Man-Man Feng, Yan Li

**Affiliations:** 1 National and Local Joint Engineering Laboratory of Traffic Civil Engineering Materials, Chongqing Jiaotong University, Chongqing 400074, China; 990020050886@cqjtu.edu.cn (X.Z.); 622160951066@mails.cqjtu.edu.cn (J.-X.H.); zczc6@cqjtu.edu.cn (C.Z.); 622160111017@mails.cqjtu.edu.cn (M.-M.F.); 2 Chongqing Tongli Expressway Maintenance Engineering Co., Ltd., Chongqing 400074, China; 622150951030@mails.cqjtu.edu.cn

**Keywords:** graphene-modified asphalt, ethylene bis(stearamide), uniform design, dispersibility, modification

## Abstract

In this study, graphene-modified asphalt (GMA) was prepared from SK-70# matrix asphalt and ethylene bis(stearamide) (EBS). Based on the uniform design method, a model was created using Data Processing System (DPS) software and First Optimization (1stOpt) software using the graphene mixing amount, EBS mixing amount, shear rate, shear time, and shear temperature as factors and using the asphalt penetration, softening point, force ductility, SHRP-PG test, and multistress creep recovery data as indices. Calculations and analysis showed that the optimal composition and preparation parameters of GMA are as follows: the graphene proportion is 20‰, the EBS proportion is 1%, the shear rate is 6000 r.p.m., the shear time is 180 min, and the shear temperature is 140 °C. The prepared GMA had a significantly improved softening point, low-temperature fracture energy, antirutting factor, and creep recovery rate, indicating that adding graphene can improve the high- and low-temperature performance of asphalt. The prepared GMA was characterized by X-ray diffraction (XRD). The dispersibility of graphene in asphalt was evaluated by fluorescence microscopy and Image-Pro Plus imaging software. The results show that graphene can exist in asphalt in a stable form, which increases the loose-layered structure of stacked asphalt or gum. The intense adsorption effect of graphene strengthens the ordered structure of asphalt. However, due to its dispersibility characteristics, some graphene exists in asphalt in clustered form. When the graphene-to-dispersant ratio approaches the optimal value, the dispersant changes the form of graphene in asphalt from irregular clusters to regular clusters and from large, distinct clusters to small, indistinct clusters. When dispersant cannot uniformly disperse graphene in asphalt, graphene clusters primarily form medium-sized grains.

## 1. Introduction

Graphene, formed by carbon atoms via sp2 electron orbital hybridization, is a beehive-shaped, two-dimensional carbon nanometer inorganic material with various superior properties. In recent years, graphene has become a focus area of scientific research [1,2,3,4,5,6,7,8,9,10,11]. Asphalt pavement is the primary form of pavement in road engineering. Based on the characteristics of the basic chemical structures of graphene and asphalt (components), graphene and asphalt share similar structures and the two should have excellent affinity [12,13]. Graphene has an enormous specific surface area and can have an intense physical adsorption effect with asphalt. Additionally, graphene is capable of physical adsorption and nonpolarized adsorption with light components and polycyclic aromatic hydrocarbons released by asphalt when heated. Under high temperature, graphene effectively suppresses the release of poisonous, harmful asphalt fumes and is environmentally friendly [14,15]. Therefore, graphene-modified asphalt (GMA) has numerous excellent properties and multiple functional groups which can significantly improve asphalt performance (such as its viscoelasticity), reduce or eliminate various asphalt pavement hazards, such as ruts, fractures, and surface wear, and reduce the cost of the entire pavement life cycle. GMA has important scientific and application value for promoting the development of high-performance and durable long-life asphalt pavement [16]. In recent years, Wang Z. et al. [17,18,19] showed that expanded graphite nanoplatelet composite-modified asphalt materials can effectively enhance the fracture recovery energy, strength, and healing capabilities of an asphalt mixture. Yao H. et al. [20] found that graphite nanoplatelet-modified asphalt can improve asphalt’s high- and low-temperature performance, its complex shear modulus, and the antirutting and waterproof capabilities of the asphalt mixture. Li Y. et al. [21] showed that when graphene oxide (GO) and asphalt were mixed, CO_2_ gas was released during GO decomposition; the GO structure was completely stripped and was scattered in asphalt to form a single layer. Huang Gang et al. [14,22,23] used expanded graphite to suppress asphalt fumes and proved that expanded graphite was infiltrated by asphalt and was stripped to form graphene platelets that were partially scattered in asphalt. Cheng I. F. et al. [24] developed a technique to produce large graphene flakes on an asphalt surface, which proved that graphene can stably exist in an asphalt medium in a single layer. The existing studies primarily focus on the modification of pavement asphalt using graphene oxide or graphene nanoplatelets to improve asphalt performance [25,26,27,28,29,30,31,32,33,34]. There is no report of research on pavement asphalt modification using graphene. 

Based on the uniform design method and using the asphalt penetration index, softening point, force ductility, SHRP-PG test, and multistress creep recovery test data as indices, this paper employed Data Processing System (DPS) and First Optimization (1stOpt) software to establish a mathematical model to investigate the material composition and preparation parameters of GMA. In addition, a microscopic analysis method and Image-Pro Plus software were applied to evaluate the dispersibility of graphene in asphalt. 

## 2. Experimental Method and Performance Evaluation

### 2.1. Materials

The matrix asphalt used in this study was SK-70# asphalt (PG64-22). Each index was tested based on the Standard Test Method of Bitumen and Bituminous Mixture for Highway Engineering (JTG E20-2011) by the Chinese Ministry of Communications [35]. The graphene was NK-1 graphene produced by the Sichuan Deyang Graphene Carbon Technology Co., Ltd., Deyang, Sichuan, China. The dispersant was ethylene bis(stearamide) (EBS) from the Malaysia Kao Company, Petaling Jaya, Malaysia. The basic solvent was trichloroethylene. The technical parameters of SK-70# asphalt, graphene, and EBS are listed in Table 1, Table 2 and Table 3, respectively.

### 2.2. Equipment and Characterization 

The shear processing of the modified asphalt was performed using a BME200L intense shear and mix emulsion machine (motor power 0.4 kw, rotational speed range 0–10,000 r.p.m.) from the Shanghai Weikang Machine Manufacturing Co., Ltd., Shanghai, China. The ultrasonic separation of the graphene mixture solution was performed using JP-040 ultrasonic equipment (ultrasonic wave power: 240 W, ultrasonic wave frequency: 40 kHz) from the Skymen Cleaning Equipment Shenzhen Co., Ltd., Shenzhen, China. The asphalt penetration index, softening point, and force ductility were measured using an SYD-2801D penetration index tester, an SYD-2806E softening point tester, and an SYD-45DBF ductility/tension tester with temperature and speed regulation from the Shanghai Changji geological instrument Co., Ltd., Shanghai, China. The asphalt rheological performance was tested with a Bohlin DSR I dynamic shear rheometer from the Malvern Panalytical Instrument Co., Ltd., Malvern, UK. The structure characterization of the modified asphalt was performed with a D8-Advance X-ray diffractometer (copper/palladium, voltage: 40 kV, current: 40 MA, test rate: 0.1 sec/step, wavelength: 1.5418 angstrom) from the Bruker Corporation, Karlsruhe, Germany. The graphene dispersion in GMA was observed using a DM6 M microscope from the Leica Microsystems Inc. Co., Ltd., Buffalo Grove, IL, USA.

### 2.3. GMA Preparation 

**Figure 1:**
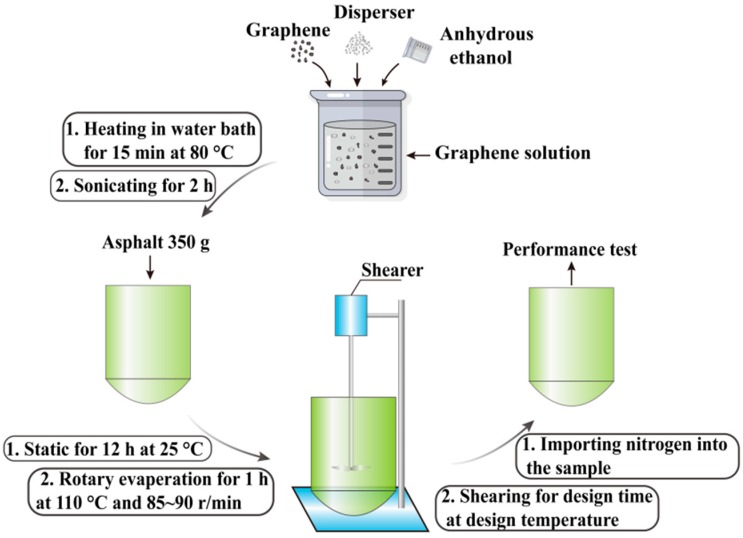
Graphene-modified asphalt preparation process.

The GMA preparation process was as follows: 

(1) The graphene and EBS were measured using an electric analytical balance (resolution: 0.0001 g, SHIMADZU Co., Tokyo, Japan) and were placed in a 1000 mL beaker. A total of 350 mL of trichloroethylene was added and mixed with a glass bar to produce a mixed solution. The mixed solution was heated in a constant temperature (80 °C) hot water bath for 15 min. Then, the opening was covered with preservative film. The mixed solution was ultrasonically processed for 2.0 h with a 5-min break every 30 min. 

(2) First, 350 g of matrix asphalt was prepared. Then, the mixed solution (after ultrasonic processing) was poured into the container filled with 350 g of matrix asphalt. The container opening was sealed with 3–4 layers of preservative film and cultured for 12 h so that the asphalt was completely dissolved in the mixed solution. The trichloroethylene in asphalt was completely removed using a rotary evaporator (from the Büchi Labortechnik AG, Uster, Switzerland) with the following parameters: oil bath temperature: 110 °C, rotational speed: 85~90 r.p.m., and evaporation time: 60 min. After the trichloroethylene was removed, the asphalt was poured into a container for the shear test to prepare the GMA with importing nitrogen into the bottom of the container continually. The GMA preparation process is shown in Figure 1.

### 2.4. Experimental Design

Uniform design is an application of the “pseudo-Monte Carlo method” in number theory. Uniform design can select a subset of typical test points from the entire set of test points, ensure the uniform distribution of test points in a test range, and reflect major features of the test system. The uniform design method is widely employed to investigate material composition and demonstrates excellent applicability and accuracy [36,37]. Therefore, in this paper, the uniform design method was employed for GMA composition design. In test design, each uniform design table has a code U_n_(q^s^). “U” represents uniform design; “n” represents n tests; “q” indicates that each factor has q levels; “s” means the table has s columns [38,39,40]. 

Five factors with significant impact (X_1_, X_2_, X_3_, X_4_, X_5_) were selected to investigate GMA composition and preparation parameters [41]. The details of these factors follow: X_1_ is the shear rate (r.p.m.); X_2_ is the shear time (min); X_3_ is the graphene proportion (‰) (mass fraction of matrix asphalt); X_4_ is the EBS proportion (%) (mass fraction of graphene); and X_5_ is the shear temperature (°C). Each factor has 10 levels, as listed in Table 4. 

Based on the factor levels in Table 4, a corresponding uniform design table and a usage table were generated to design combinations of test parameters. The obtained test parameter combinations are listed in Table 5. 

Based on the preparation parameters of each test group in Table 5, the GMA was prepared and subsequent performance tests were performed.

### 2.5. Performance Evaluation and Microanalysis

The GMA pavement performance was analyzed via its penetration index, softening point, and force ductility index. An SHRP-PG test and a multistress creep recovery test were performed to analyze the viscoelasticity of GMA. The GMA structure was characterized via XRD (from the Bruker Corporation, Karlsruhe, Germany) and a fluorescence microscope (from the Leica Microsystems Inc. Co., Ltd., Buffalo Grove, IL, USA).

## 3. Results and Discussion

### 3.1. Indices Data Analysis

**Figure 2:**
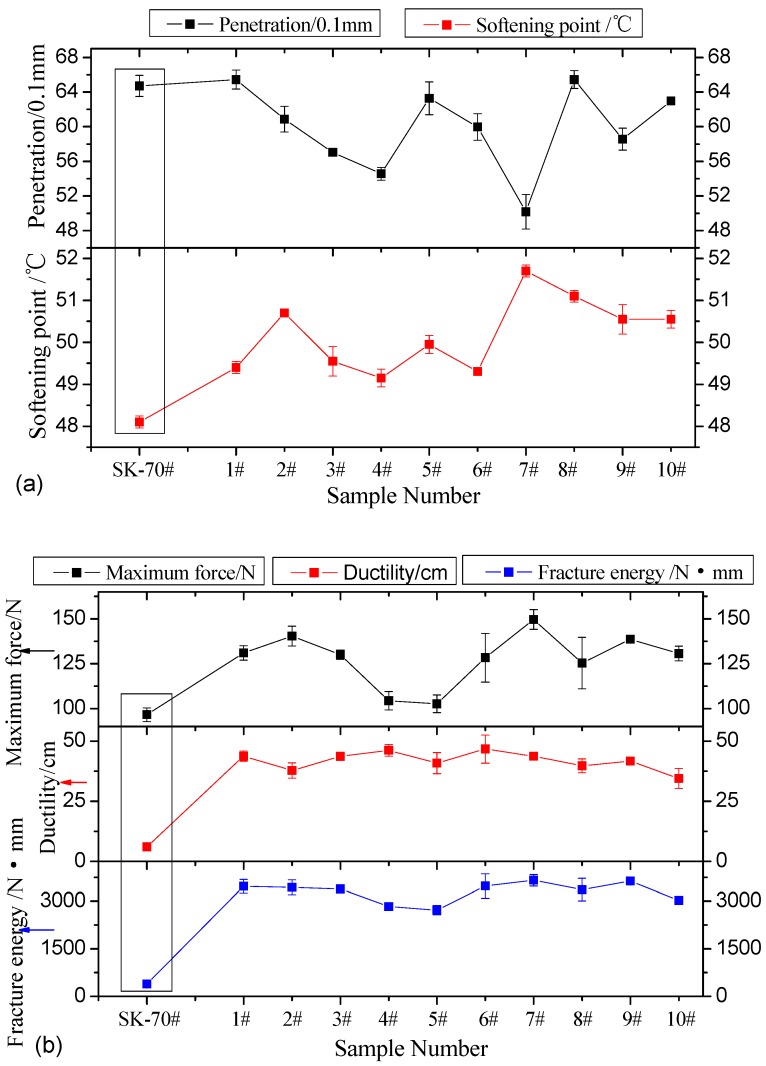
(**a**,**b**) Conventional asphalt performance index test results.

The penetration index represents the asphalt thickness at the test temperature, which reflects asphalt’s rheological performance to some extent [42,43]. The test conditions were as follows: the water bath was at a constant temperature of 25 °C, the standard penetration load was 100 g, and the penetration time was 5 s. The softening point is the critical temperature at which asphalt changes from a solid state to a liquid, which reflects the temperature response performance of the asphalt material [44]. The ductility reflects asphalt’s deformation capability at a specified temperature and its stretch rate before it is stretched to rupture [45,46]. In this study, the force ductility test environment was as follows: the water bath was at a constant temperature of 5 °C, and the stretch rate was 5 cm/min. Three indices were obtained during the asphalt specimen tensile process: force, ductility, and fracture energy (the integral of force and ductility). The test results for the asphalt indices are shown in Figure 2a,b.

The penetration test result in Figure 2 shows that after graphene was added, except for test groups 1 and 8, the asphalt penetration indices decreased. Test group 6 had the minimum penetration at 5.02 mm. Test groups 1 and 8 had the maximum penetration indices, at 6.54 mm. The penetration test results indicate that the graphene addition hardened the asphalt overall, improving its high-temperature performance. The softening point test results show that after adding graphene, the asphalt softening points in all test groups increased. Test group 7 had the maximum softening point at 51.7 °C. The softening point test results suggest that adding graphene improves asphalt’s high-temperature performance. The force ductility test results show that after adding graphene, the maximum ductility force, ductility, and fracture energy improved significantly. Test group 7 had the maximum ductility force at 150.0 N; test group 6 had the maximum ductility at 46.70 cm; and test group 9 had the maximum fracture energy at 3633.0 N·mm. The force ductility test results demonstrate that adding graphene significantly improves asphalt’s low-temperature performance. To summarize, graphene addition improves both the high- and low-temperature performance of asphalt. The optimal material composition and preparation parameters for preparing GMA are similar to the design parameters of test groups 6–9. 

### 3.2. DSR Test

The rheological parameter of graphene asphalt was tested using the Dynamic Shear Rheological test (DSR) proposed by the Strategic Highway Research Project (SHRP) in the United States to characterize viscoelastic energy and evaluate the high- and low-temperature performance and the antifatigue performance of asphalt [47]. 

#### 3.2.1. SHRP-PG Test

**Figure 3:**
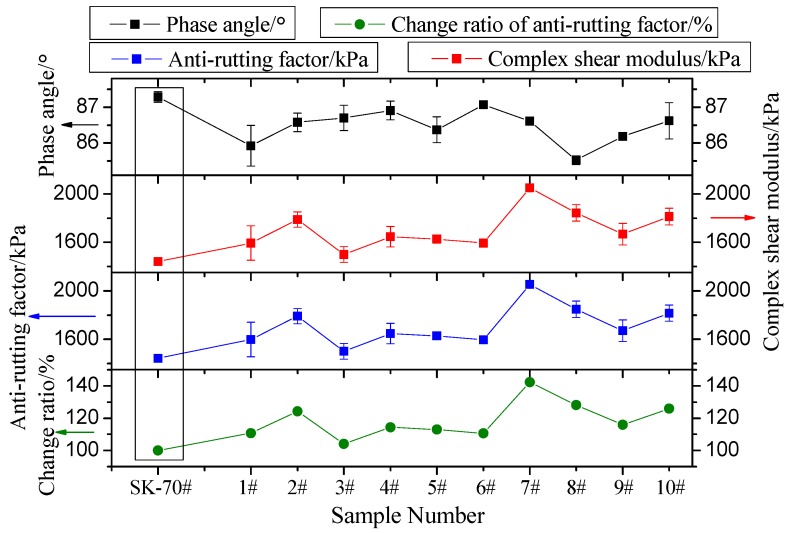
SHRP-PG test results (64 °C): The changing trends of phase angle, complex shear modulus, antirutting factor, and change ratio of antirutting factor are shown separately. The change ratio of antirutting factor is that the antirutting factor of each test group is divided by the antirutting factor of SK-70# base asphalt.

The SHRP-PG evaluates the high-temperature performance indices of asphalt cement material. The test reflects two important parameters of asphalt’s viscoelasticity: the complex shear modulus G* and the phase angle δ. The complex shear modulus G* is the ratio of the maximum shear stress and the maximum shear strain in the SHRP-PG classification test. The complex shear modulus G* represents the overall resistance of a material under repeated shear deformation, which includes the elastic modulus G′ and the viscous modulus G″. The elastic modulus is given by G′ = G*cosδ, which reflects the asphalt energy stored and released during shear deformation. The viscous modulus is given by G″ = G*sinδ, which represents the dissipated energy in the form of heat due to internal friction during the asphalt shear process. G*sinδ is defined as the antirutting factor, which represents the capability of asphalt cement material to resist permanent deformation under high temperature [48,49]. In this study, the test temperature was 64 °C; the diameter of the smooth metal plate was 25 ± 0.05 mm; the gap between the test plate and the roof was 1 ± 0.05 mm; and the test frequency was 10 rad/s. The test results are shown in Figure 3.

Figure 3 shows that after graphene is added, the GMA phase angle decreases to some extent, while the complex shear modulus and the antirutting factor improve to some extent. Test groups 7, 8, and 10 had the most significant improvement in antirutting factor (42.4%, 28.2%, and 25.9%, respectively). It can be inferred that adding graphene improves asphalt’s high-temperature stability. The optimal proportions of graphene and dispersant for graphene asphalt preparation is similar to the material design parameters for test groups 7, 8, and 10.

#### 3.2.2. Multistress Creep Recovery (MSCR) Test

**Figure 4:**
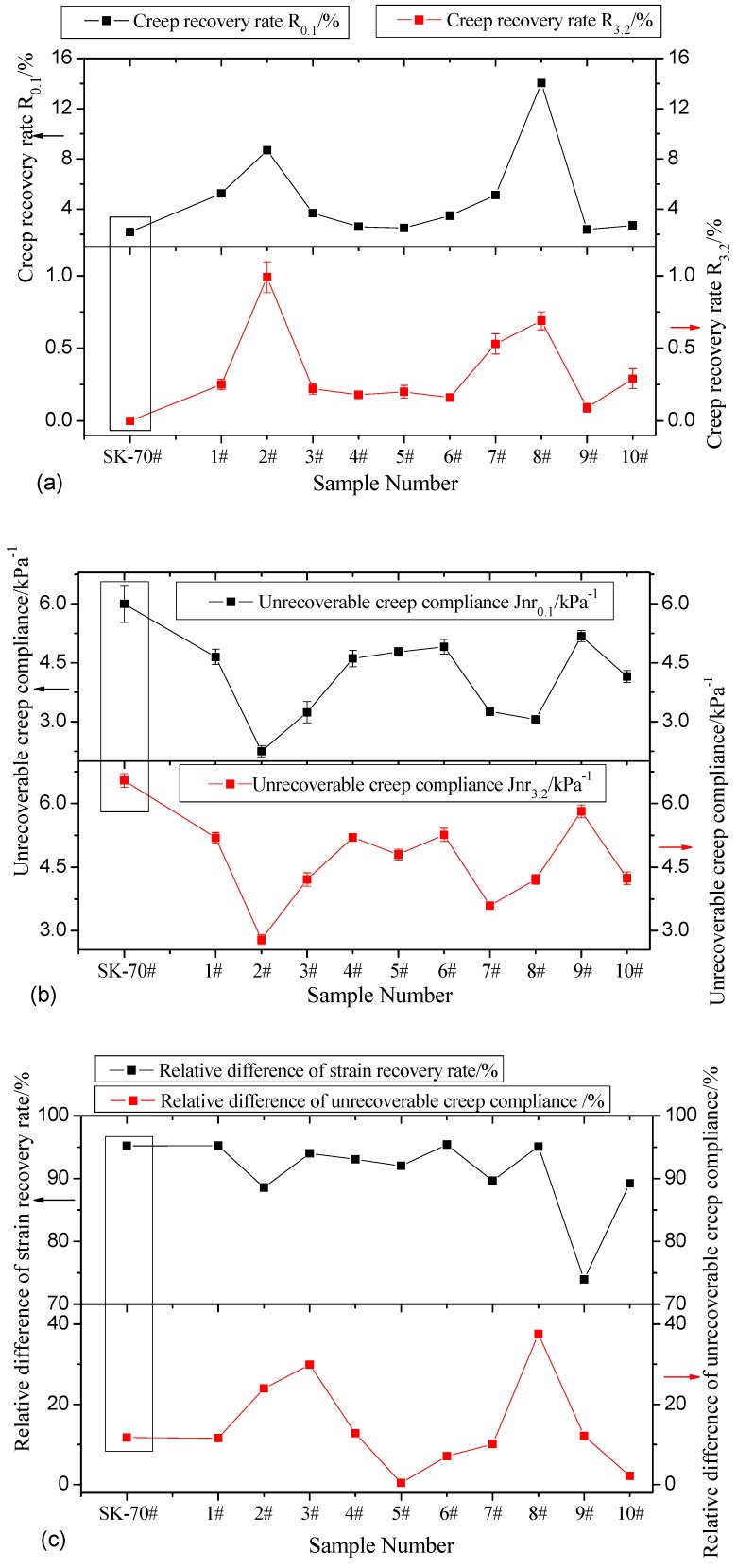
(**a**–**c**) Creep recovery test result.

Repeated multistress creep recovery tests were performed to further evaluate GMA’s high-temperature stability. The test temperature was based on the SHRP-PG classification test result and the AASHTO T350-14 specification [50,51,52]. First, a 100 Pa shear stress was applied for 100 s. Then, while the 100 Pa shear stress was applied, cyclic loading (1 s loading and 9 s unloading) was repeated 10 times. Next, a 3200 Pa shear stress was applied to repeat the above process. The entire test included 30 cycles and took 300 s. The delayed elasticity recovery capability of GMA was evaluated via the recovery rate R and the unrecoverable creep compliance Jnr. The test results are shown in Figure 4a–c. 

Figure 4 shows that compared with matrix asphalt, GMA’s creep recovery rate under 0.1 kPa shear stress and its creep recovery rate under 3.2 kPa of shear stress improve to some extent, indicating that the addition of graphene improves the asphalt’s viscoelastic recovery capability. Test groups 8, 2, and 7 have superior creep recovery rates at 14.04%, 8.68%, and 5.12%, respectively, which are 6.41 times, 3.96 times, and 2.34 times greater than those for matrix asphalt. In the 3.2 kPa creep recovery test, matrix asphalt has almost no creep recovery, while groups 2, 8, and 7 have improved creep recovery rates at 0.99%, 0.69%, and 0.53%, respectively. The optimal parameters for GMA are similar to the parameters for groups 2, 7, and 8. 

To summarize, based on a test of three major indices and the DSR test result, the optimal material composition and parameters for GMA preparation are similar to the design parameters for test groups 7 and 8. 

### 3.3. Determining the Optimum Mixing Ratio

In this paper, Data Processing System (DPS) analysis software (Version DPSv17.10) and First Optimization (1stOpt) software (Version 7.0) are employed to calculate the optimal material composition for GMA preparation. DPS is a data processing system that integrates functions such as numeric calculation, statistical analysis, model simulation, drawing, and table generation [53,54]. 1stOpt is general-purpose numerical optimization simulation software with various classical and modern optimization algorithms that produce accurate solutions for nonlinear optimization problems [55,56]. Because conventional least square multiple linear regression and progressive regression analysis methods cannot meet the requirement of multiparameter and nonlinear test design modeling, three regression models, “partial least square quadratic polynomial”, “partial least square quadratic term”, and “partial least square interaction term”, are employed in this paper. DPS software and 1stOpt software are employed to find the optimal GMA material composition. 

The interdependency of three force ductility test parameters (force, ductility, and fracture energy) in modeling results in multiple colinearity and an unstable calculation result, which impacts the model creation significantly. Therefore, five indices (penetration Y_1_, fracture energy Y_2_, softening point Y_3_, 64 °C antirutting factor Y_4_, and 0.1 kPa creep recovery rate Y_5_) are selected to create the regression model for calculation and analysis. During modeling, based on the PRESS statistics after data standardization and a declining trend in the sum of the squared errors, the determinant coefficient R^2^ is defined as the major criterion to evaluate the regression model’s effectiveness. A larger determinant coefficient indicates better equation fitting. The relationship between the number of latent variables and the determinant coefficient in three regression models calculated by DPS software is given in Table 6.

Table 6 shows that as the number of latent variables increases, the determinant coefficient R^2^ gradually increases. When the number of latent variables is 5, the determinant coefficient R^2^ reaches its maximum level. This means the regression method created using the partial least square method has a higher degree of fitting, and the model is closer to the actual situation and reliable. The coupling of five factors in the model leads to significant changes in GMA performance. The equation groups of three regression models are given in Table 7.

Based on Table 6, a comparison of the determinant coefficients in the three regression models shows that the partial least square quadratic polynomial regression model has small Y_3_ and Y_5_ determinant coefficients and a relatively low degree of fitting. Therefore, this model is excluded. By comparison, partial least square interaction term regression and partial least square quadratic term regression have large determinant coefficients and regression models with higher degrees of fitting. Therefore, these two models are employed to find the optimal solution for GMA material composition and preparation parameters.

Table 7 shows that all three regression models are nonlinear. Considering that there are multiple solutions in the actual calculation, 1stOpt software is employed in the regression model for optimization. The results are listed in Table 8. 

Based on Table 8, the optimal graphene asphalt material composition and preparation parameters are obtained to prepare GMA for performance tests and verification. The results are listed in Table 9.

In Table 9, change rate is that the test value of GMA is divided by that of SK-70# matrix asphalt in the same test item. Table 9 shows that compared with matrix asphalt, the prepared GMA has a smaller penetration and a significantly higher softening point, force ductility force, ductility, fracture energy, 64 °C anti-rutting factor, and 0.1 kPa creep recovery rate. In tests B-1, B-2, and B-3, compared with matrix asphalt, fracture energy values at low temperature improve by 940.93%, 813.70%, and 766.21%, respectively; 64 °C anti-rutting factors improve by 45.54%, 13.92%, and 0.05%, respectively; and creep recovery rates improve by 824.20%, 299.54%, and 262.10%, respectively.

To summarize, in three optimal formulae, compared with matrix asphalt, the prepared GMA has a smaller penetration index, and the asphalt is hardened. Additionally, high- and low-temperature performance and delayed elasticity recovery improve significantly. This is likely because some of the graphene has intercalated in the asphalt, which causes a strengthening effect. Test group B-1 had the most significant performance improvement; hence, test B-1 parameters are selected as optimal GMA mix parameters: the high-speed shear rate is 6500 r.p.m.; the shear time is 180 min; the graphene proportion is 20‰; the EBS proportion is 1%; and the shear temperature is 140 °C. 

### 3.4. Textural Characterization

#### 3.4.1. XRD Test

**Figure 5:**
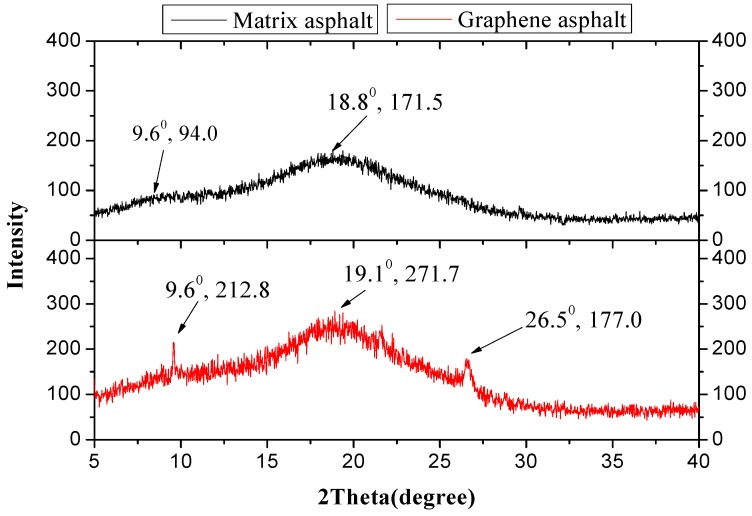
XRD test spectrum of matrix asphalt and GMA.

In the XRD test, the material under analysis undergoes X-ray diffraction to obtain a diffraction spectrum, which is used to investigate useful material characteristics such as crystal structure and elemental composition [57]. XRD analysis was performed on the SK-70# matrix asphalt and the BEST-1 GMA; the results are shown in Figure 5. 

Based on Figure 5, the matrix asphalt spectrum shows the most intense peak is at approximately 2θ = 18.8°. Based on Bragg’s law, 2dsinθ = nλ, the interplanar spacing is d_1_ = 0.472 nm and there is an extremely weak peak at 2θ = 9.6°; the interplanar spacing is d_2_ = 0.921 nm, which is a loose-layered structure of stacked asphalt or gum. The GMA spectrum shows peaks at 2θ = 9.6° and 2θ = 19.1°; the interplanar spacing values are d_3_ = 0.921 nm and d_4_ = 0.467 nm, respectively; there is a new peak at approximately 2θ = 26.5°, which is the graphene characteristic peak [57] with a strength of 177 cps and an interplanar spacing of d_5_ = 0.336 nm. The spectrum demonstrates the existence of graphene in asphalt. After graphene is added, the strength of the asphalt or gum characteristic peak increases to some extent, which means that graphene increases its loose-layered structure of stacked asphalt or gum. Peak spacing decreases to some extent, indicating that the intense adsorption effect of graphene enhances the ordered structure of asphalt. 

#### 3.4.2. Microscope Test

**Figure 6:**
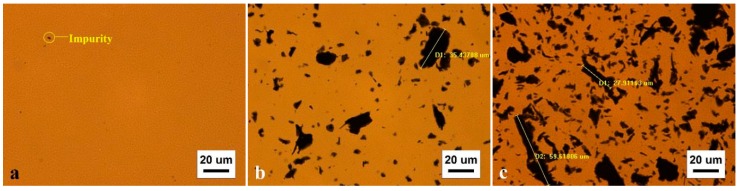

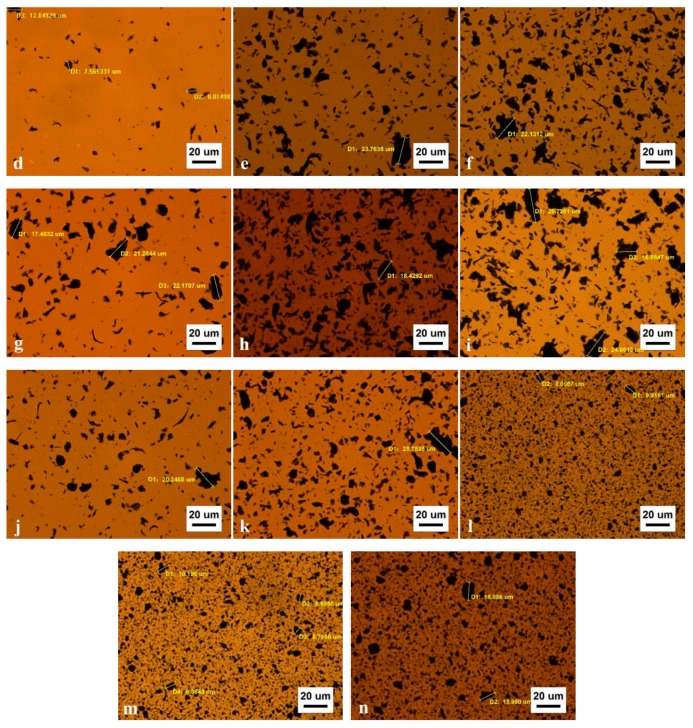
Microscopy test results ((**a**) is the test result of SK-70# matrix asphalt; (**b**–**k**) are the test results of GMA uniform design groups 1–10; (**l**–**n**) are the test results of B-1~B-3 GMA).

Due to its advantages, including convenient operation and easy sample preparation, the fluorescence microscope has become a widely used tool to observe micromorphology of materials, and has been used in asphalt characterization [58]. In this paper, SK-70# matrix asphalt, GMA in uniform design test groups 1–10, and GMA in the three groups with optimal admixtures were observed using a fluorescence microscope. The test results are shown in Figure 6. 

A comparison of matrix asphalt in Figure 6a and GMA in Figure 6b–k shows that various forms of black substances are observed in all graphene asphalt samples. As graphene is a nanometer material, observation under a normal fluorescence microscopy condition is very difficult. If graphene is distributed evenly in asphalt under the effect of stearic amide dispersant, then graphene asphalt topography observed in a fluorescence microscopic image with 500× magnification should essentially be identical to matrix asphalt topography. However, the actual observation shows that graphene asphalt contains a large amount of a black substance. Graphene has an extremely large specific surface area and a strong interlayer force, and therefore is very difficult to distribute completely uniformly [59,60,61]. Because the XRD test proves the stable existence of graphene in asphalt, this black substance should be graphene clusters. EBS cannot distribute graphene evenly in asphalt. 

Figure 6l–n show that compared with materials with other compositions, the graphene clusters in the GMA prepared with the optimal material composition obtained from modeling have more regular, spherical shapes. This means that with the optimal graphene and dispersant mixture ratio, the dispersant changes the graphene topography in asphalt; the graphene clusters evolve from large, distinct, irregular shapes to small, indistinct, regular shapes. 

**Figure 7:**
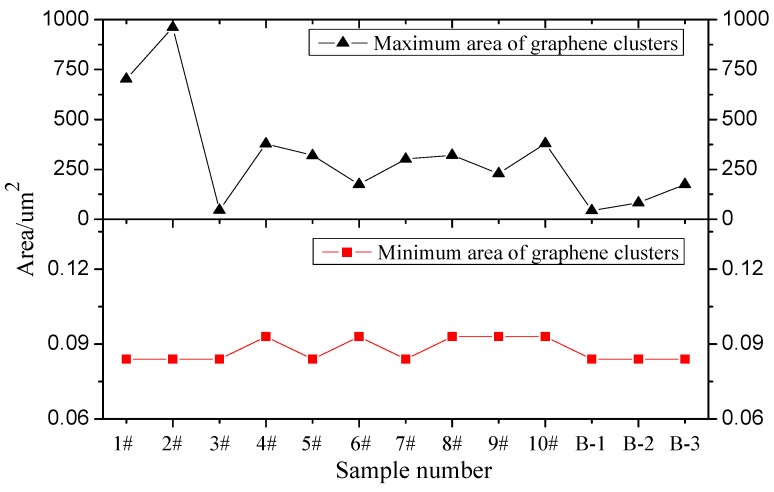
Variation trend of graphene cluster max and min areas.

**Figure 8:**
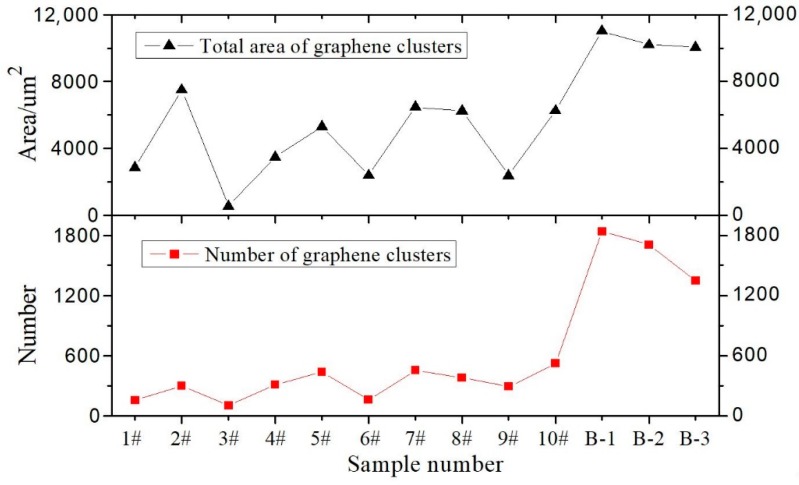
Variation trend of graphene cluster total area and quantity.

**Figure 9:**
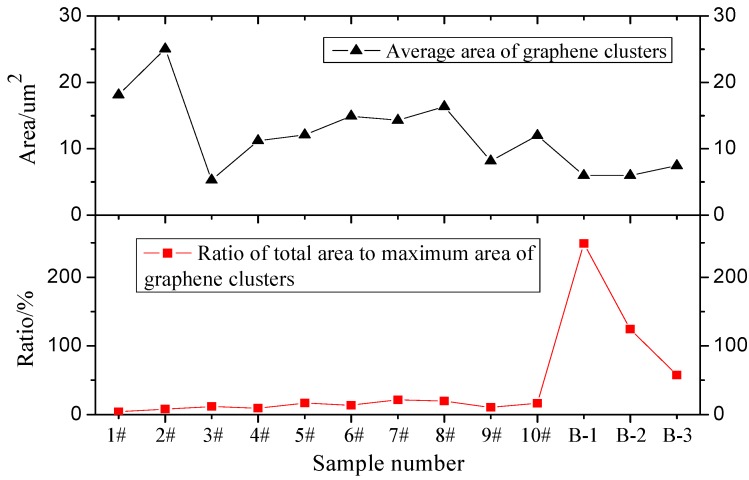
Variation trend of graphene cluster average area and ratio of total area to maximum area of graphene clusters.

Image-Pro Plus is widely used microscopy image analysis software with accurate and reliable image analysis results. In recent years, image analysis has been applied extensively in civil engineering research [62,63,64]. In this paper, Image-Pro Plus software is employed to analyze GMA images and obtain test group parameters, such as the number of graphene clusters, the maximum area, minimum area, total area, cluster average area, total area, and ratio to maximum cluster area. The results are shown in Figure 7, Figure 8 and Figure 9. 

Figure 7 and Figure 8 show that the dispersant has significantly different graphene dispersion effects in different test groups (i.e., the graphene distribution in asphalt is affected by differences in parameters including the dispersant and graphene mix ratio, shear rotating speed, shear time, and shear temperature). In different test groups, the graphene clusters have similar minimum areas. Although Figure 7 shows the maximum graphene cluster areas in different test groups differ significantly, such differences reflect differences between individual graphene cluster areas and cannot represent the general variation pattern of graphene clusters in the test groups. Therefore, the maximum and minimum graphene cluster areas have no comparative significance. In the optimal parameter solution, the optimal graphene asphalt material composition and preparation parameters are based on test group B-1. Figure 8 shows a larger graphene cluster total area and more clusters. Figure 9 shows a small graphene cluster average area with a significantly larger total area and maximum area ratio than other test groups. This means this test group has properly distributed graphene in asphalt. 

**Figure 10:**
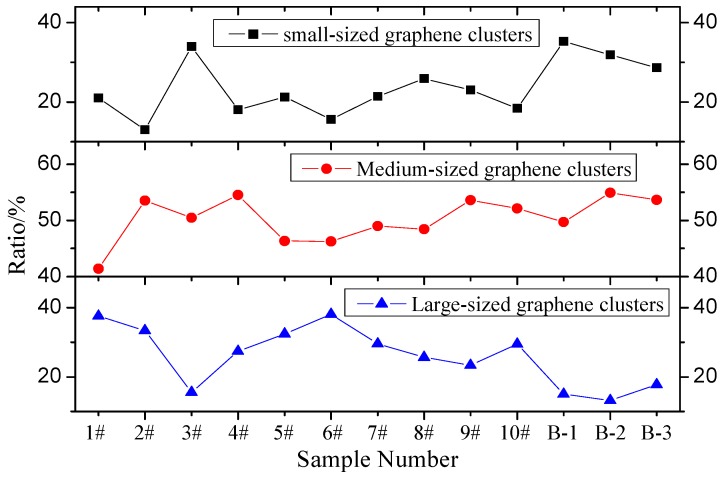
Variation trend of different categories of graphene clusters.

**Figure 11:**
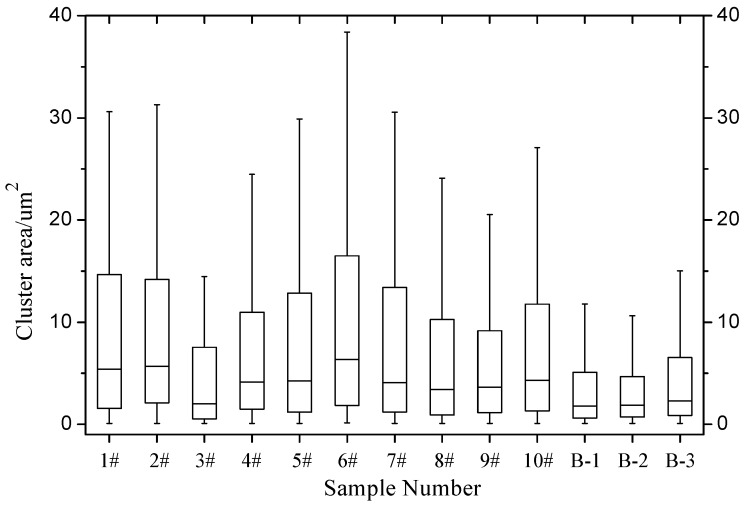
Box plot of graphene cluster area.

Based on the above graphene cluster characteristics, clusters in images are divided into three categories based on dimension: fine clusters, medium clusters, and coarse clusters. The fine cluster area is less than 1 µm^2^; the medium cluster area is between 1 µm^2^ and 10 µm^2^; the coarse cluster area is larger than 10 µm^2^. Based on these categories, the graphene cluster distribution patterns in various test groups scattered by EBS are shown in Figure 10 and Figure 11. 

Figure 10 and Figure 11 show that in different test groups, the coarse- and fine-grain proportions of graphene clusters in graphene asphalt vary significantly. In contrast, the medium grain ratio has a small variation and is essentially stable. This means when dispersant cannot distribute graphene evenly in asphalt, the majority of graphene clusters in asphalt are medium-sized. 

The performance comparison shows that test group B-1 had the smallest quartile and median among all test groups. Test group B-1 had the highest proportion of fine graphene clusters, a small proportion of coarse clusters, the largest total cluster area, clusters with small dimension, and the maximum softening point, low temperature ductility fracture energy, antirutting factor, and 0.1 kPa creep recovery rate at 58.6 °C, 4035.7 N·mm, 2099.00 kPa, and 20.24%, respectively. Again, this means graphene in this test group is distributed properly in asphalt, resulting in a significant improvement in macroscopic asphalt performance.

To summarize, in EBS-based GMA, when the graphene and dispersant proportions and corresponding preparation parameters are optimal, graphene is distributed properly in asphalt, which significantly improves the softening point, low-temperature ductility fracture energy, antirutting factor, and creep recovery rate of the material.

## 4. Conclusions

(1) A method for calculating the optimal parameters of GMA and a process to prepare GMA were proposed. For EBS-based GMA, the optimal parameters are as follows: the graphene proportion is 20‰; the EBS proportion is 1%; the high-speed shear rate is 6000 r.p.m.; the shear time is 180 min; the shear temperature is 140 °C. The prepared GMA had a significantly improved softening point, low temperature fracture energy, antirutting factor, and creep recovery rate.
(2) The prepared GMA had a softening point of 58.6 °C, a low-temperature ductility force of 168.0 N, low-temperature ductility of 42.54 mm, low-temperature fracture energy of 2099 N·mm, and a 0.1 kPa creep recovery rate of 20.24%. Compared with SK-70# matrix asphalt, the performance of GMA was significantly improved.
(3) Graphene can exist in an asphalt medium in a stable form, and some graphene in asphalt is in the form of clusters. When the graphene and dispersant composition is close to the optimal ratio, the dispersant changes the form of graphene in asphalt from irregular clusters to regular clusters and from distinct, large clusters to indistinct, small clusters. When the graphene distribution in asphalt is closer to the ideal situation, graphene asphalt has improved high- and low-temperature performance. When the dispersant cannot distribute graphene evenly in asphalt, the majority of graphene clusters in asphalt are medium-sized.
(4) Although EBS is used in this study, graphene is still not distributed evenly in asphalt in the form of flakes but is in the form of small clusters. Methods to ideally disperse or intercalate graphene in asphalt to substantially improve asphalt performance require further investigation.

## Figures and Tables

**Table 1:** Parameters of SK-70# asphalt.

Test Item		Test Result	Technology Index	Test Method
penetration (25 °C, 5 s, 100 g)/0.1 mm		64.70	60.0~80.0	T0604
ductility (15 °C, 5 cm/min)/cm		103.00	≥100.0	T0605
softening point/°C		48.10	≥45.0	T0606
density (15 °C)g/cm^3^		1.21	actual measurement	T0603
wax content/%		2.04	≤2.2	T0615
dynamic viscosity(60 °C)/Pa·s		197	≥180	T0620
flash point/°C		315	≥260	T0611
after RTFOT	mass change/%	−0.18	≤±0.8	T0610
	residual penetration ratio/%	63.50	≥61.0	T0604
	10 °C ductility/cm	8.60	≥6.0	T0605

**Table 2:** Parameters of graphene NK-1.

Parameter	Index
graphene layers/thickness	1–3, monolayer rate >80%
ash content/%	<3.0
specific surface area/m²/g	110.0
film electrical conductivity/S/cm	550.0
flake diameter (D50)/um	7.0~12.0
flake diameter (D90)/um	11.0~15.0
appearance	Black-grey powder
bulk density/g/mL	0.01~0.02
water content/%	<2.0

**Table 3:** Parameters of dispersant ethylene bis(stearamide).

Parameter	Index
appearance	White powder
initial melting point/°C	141.0~146.0
total amine/mg KOH/g	≤3.0
color value	≤5.0
acid value/mg KOH/g	≤7.0
fineness degree/mesh	600
heating decrement/%	≤0.5
flash point/°C	≥28.0

**Table 4:** Test design factor levels.

Factor	Level									
	1	2	3	4	5	6	7	8	9	10
X_1_/r.p.m.	2000	2500	3000	3500	4000	4500	5000	5500	6000	7000
X_2_/min	30	30	60	60	90	90	120	120	180	180
X_3_/‰	2	4	6	8	10	12	14	16	18	20
X_4_/%	1	2	3	4	5	6	7	8	9	10
X_5_/℃	110	110	120	120	130	130	140	140	150	150

**Table 5:** Test combinations design table.

Test #	Factor				
	X_1_/r.p.m.	X_2_/min	X_3_/‰	X_4_/%	X_5_/°C
1#	2000	60	8	5	150
2#	2500	90	16	10	140
3#	3000	180	2	4	130
4#	3500	30	10	9	120
5#	4000	60	18	3	110
6#	4500	120	4	8	150
7#	5000	180	12	2	140
8#	5500	30	20	7	130
9#	6000	90	6	1	120
10#	7000	120	14	6	110

**Table 6:** The number of latent variables versus the determinant coefficient.

The Number of Latent Variables	Partial Least Square Quadratic Polynomial Regression Determinant Coefficient R^2^					Partial Least Square Quadratic Term Regression Determinant Coefficient R^2^					Partial Least Square Interaction Term Regression Determinant Coefficient R^2^				
	Y_1_	Y_2_	Y_3_	Y_4_	Y_5_	Y_1_	Y_2_	Y_3_	Y_4_	Y_5_	Y_1_	Y_2_	Y_3_	Y_4_	Y_5_
1	0.720	0.274	0.262	0.294	0.001	0.740	0.387	0.278	0.216	0.009	0.694	0.346	0.326	0.363	0.001
2	0.923	0.336	0.401	0.317	0.591	0.777	0.391	0.795	0.714	0.424	0.911	0.374	0.559	0.470	0.646
3	0.944	0.424	0.658	0.764	0.631	0.813	0.760	0.822	0.732	0.882	0.912	0.651	0.663	0.733	0.652
4	0.961	0.688	0.701	0.787	0.699	0.843	0.921	0.921	0.825	0.941	0.973	0.789	0.820	0.849	0.669
5	0.965	0.864	0.796	0.835	0.749	0.976	0.941	0.943	0.876	0.973	0.977	0.922	0.881	0.906	0.879

**Table 7:** Equation data of regression fitting model.

Regression Model	Partial Least Square Quadratic Polynomial Regression Model	Partial Least Square Quadratic Term Regression Model	Partial Least Square Interaction Term Regression Model
regression equation of penetration	Y_1_ = 69.065 + 5.02 × 10^−4^ × X_1_ + 0.248 × X_2_ + 0.236 × X_3_ − 2.019 × X_4_ − 0.291 × X_5_ − 2.58 × 10^−4^ × X_2_^2^ + 1.99 × 10^−2^ × X_3_^2^ − 8.66 ×10^−2^ × X_4_^2^ + 2.21 × 10^−3^ × X_5_^2^ – 5 × 10^−6^ × X_1_ × X_2_ + 6.3 × 10^−5^ × X_1_ × X_3_ + 1.36 × 10^−4^ X_1_ × X_4_ − 3.2 × 10^−5^ × X_1_ × X_5_ − 1.24 × 10^−3^ × X_2_ × X_3_ + 6.94 × 10^−3^ × X_2_ × X_4_ − 1.7 × 10^−3^ × X_2_ × X_5_ + 1.5 × 10^−2^ × X_3_ × X_4_ − 5.54 × 10^−3^ × X_3_ × X_5_ + 1.33 × 10^−2^ × X_4_ × X_5_	Y_1_ = 145.630 − 6.78 × 10^−3^ × X_1_ + 9.45 × 10^−2^ × X_2_ − 1.488 × X_3_ + 2.012 × X_4_ − 1.153 × X_5_ + 1 × 10^−6^ × X_1_^2^ − 6.22 × 10^−4^ × X_2_^2^ + 7.31 × 10^−2^ × X_3_^2^ − 0.189 × X_4_^2^ + 4.47 × 10^−3^ × X_5_^2^	Y_1_ = 12.794 + 5.05 × 10^−3^ × X_1_ + 0.266 × X_2_ + 1.397 × X_3_ − 4.966 × X_4_ + 0.434 × X_5_ – 5 × 10^−6^ × X_1_ × X_2_ + 6.9 × 10^−5^ × X_1_ × X_3_ + 1.91 × 10^−4^ × X_1_ × X_4_ − 4.9 × 10^−5^ × X_1_ × X_5_ − 1.39 × 10^−3^ × X_2_ × X_3_ + 1.13 × 10^−2^ × X_2_ × X_4_ − 2.39 × 10^−3^ × X_2_ × X_5_ + 9.34 × 10^−3^ × X_3_ × X_4_ − 1.09 × 10^−2^ × X_3_ × X_5_ + 2.39 × 10^−2^ × X_4_ × X_5_
regression equation of fracture energy	Y_2_ = −7.782+1.16 × 10^−2^ × X_1_ + 1.081 × X_2_ − 8.445 × X_3_ − 15.546 × X_4_ + 4.022 × X_5_ + 2 × 10^−6^ × X_1_^2^ + 5.57 × 10^−4^ × X_2_^2^ + 4.82 × 10^−2^ × X_3_^2^ + 0.233 × X_4_^2^ − 9.80 × 10^−3^ × X_5_^2^ − 8 × 10^−6^ × X_1_ × X_2_ − 1.67 × 10^−4^ × X_1_ × X_3_ − 7.05 × 10^−4^ × X_1_ × X_4_ − 1.37 × 10^−4^ × X_1_ × X_5_ + 1.92 × 10^−3^ × X_2_ × X_3_ − 5.54 × 10^−3^ × X_2_ × X_4_ − 6.86 × 10^−3^ × X_2_ × X_5_ + 0.349 × X_3_ × X_4_ + 4.28 × 10^−2^ × X_3_ × X_5_ + 7.85 × 10^−2^ × X_4_ × X_5_	Y_2_ = −465.825 − 5.36 × 10^−2^ × X_1_ + 0.209 × X_2_ + 0.885 × X_3_ − 9.127 × X_4_ + 12.342 × X_5_ + 7 × 10^−6^ × X_1_^2^ + 5.2 × 10^−5^ × X_2_^2^ − 1.56 × 10^−2^ × X_3_^2^ + 0.616 × X_4_^2^ − 4.19 × 10^−2^ × X_5_^2^	Y_2_ = 112.752 + 2.48 × 10^−2^ × X_1_ + 0.944 × X_2_ − 8.115 × X_3_ − 10.189 × X_4_ + 1.410 × X_5_ + 1.3 × 10^−5^ × X_1_ × X_2_ − 2.61 × 10^−4^ × X_1_ × X_3_ − 9.33 × 10^−4^ × X_1_ × X_4_ − 1.16 × 10^−4^ × X_1_ × X_5_ + 5.59 × 10^−3^ × X_2_ × X_3_ − 1.51 × 10^−2^ × X_2_ × X_4_ − 5.56 × 10^−3^ × X_2_ × X_5_ + 0.361 × X_3_ × X_4_ + 5.25 × 10^−2^ × X_3_ × X_5_ + 7.08 × 10^−2^ × X_4_ × X_5_
regression equation of softening point	Y_3_ = 41.772 + 1.71 × 10^−4^ × X_1_ + 1.13 × 10^−2^ × X_2_ − 0.106 × X_3_ − 0.142 × X_4_ + 8.69 × 10^−2^ × X_5_ + 1.2 × 10^−5^ × X_2_^2^ + 1.88 × 10^−3^ × X_3_^2^ − 2.17 × 10^−3^ × X_4_^2^ − 2.02 × 10^−4^ × X_5_^2^ + 1 × 10^−6^ × X_1_ × X_2_ + 4 × 10^−6^ × X_1_ × X_3_ − 3 × 10^−6^ × X_1_ × X_5_ + 8.6 × 10^−5^ × X_2_ × X_3_ − 4.69 × 10^−4^ × X_2_ × X_4_ − 8 × 10^−5^ × X_2_ × X_5_ + 6.23 × 10^−3^ × X_3_ × X_4_ + 6.17 × 10^−4^ × X_3_ × X_5_ + 7.55 × 10^−4^ × X_4_ × X_5_	Y_3_ = 19.483 − 8.74 × 10^−4^ × X_1_ − 8.32 × 10^−4^ × X_2_ + 0.129 × X_3_ − 0.348 × X_4_ + 0.467 × X_5_ + 2.4 × 10^−5^ × X_2_^2^ − 2.08 × 10^−3^ × X_3_^2^ + 2.6 × 10^−2^ × X_4_^2^ − 1.72 × 10^−3^ × X_5_^2^	Y_3_ = 45.499 + 3.88 × 10^−4^ × X_1_ + 1.39 × 10^−3^ × X_2_ − 8.17 × 10^−2^ × X_3_ − 3.67 × 10^−2^ × X_4_ + 2.26 × 10^−2^ × X_5_ + 1 × 10^−6^ × X_1_ × X_2_ + 1 × 10^−6^ × X_1_ × X_3_ − 8 × 10^−6^ × X_1_ × X_4_ − 2 × 10^−6^ × X_1_ × X_5_ + 1.98 × 10^−4^ × X_2_ × X_3_ − 7.9 × 10^−4^ × X_2_ × X_4_ + 2 × 10^−6^ × X_2_ × X_5_ + 5.77 × 10^−3^ × X_3_ × X_4_ + 8.89 × 10^−4^ × X_3_ × X_5_ + 2.43 × 10^−4^ × X_4_ × X_5_
regression equation of anti-rutting factor	Y_4_ = 903.586 + 2.12 × 10^−4^ × X_1_ − 1.517 × X_2_ + 1.279 × X_3_ + 4.146 × X_4_ + 5.787 × X_5_ + 3 × 10^−6^ × X_1_^2^ + 2.48 × 10^−3^ × X_2_^2^ + 5.2 × 10^−2^ × X_3_^2^ − 0.291 × X_4_^2^ − 7.68 × 10^−3^ × X_5_^2^ + 2.44 × 10^−4^ × X_1_ × X_2_ + 4.46 × 10^−4^ × X_1_ × X_3_ + 1.98 × 10^−4^ × X_1_ × X_4_ − 2.54 × 10^−4^ × X_1_ × X_5_ + 3.84 × 10^−2^ × X_2_ × X_3_ − 0.114 × X_2_ × X_4_ + 6.55 × 10^−3^ × X_2_ × X_5_ + 0.361 × X_3_ × X_4_ + 3.92 × 10^−2^ × X_3_ × X_5_ − 2.03 × 10^−2^ × X_4_ × X_5_	Y_4_ = −2748.009 − 0.116 × X_1_ − 0.855 × X_2_ + 55.224 × X_3_ − 50.766 × X_4_ + 63.364 × X_5_ + 1.8 × 10^−5^ × X_1_^2^ + 7.32 × 10^−3^ × X_2_^2^ − 1.742 × X_3_^2^ + 4.757 × X_4_^2^ − 0.228 × X_5_^2^	Y_4_ = 1256.714 + 5.72 × 10^−3^ × X_1_ − 3.325 × X_2_ + 1.769 × X_3_ + 21.236 × X_4_ + 0.849 × X_5_ + 2.97 × 10^−4^ × X_1_ × X_2_ − 1.4 × 10^−5^ × X_1_ × X_3_ − 6.69 × 10^−4^ × X_1_ × X_4_ + 1.25 × 10^−4^ × X_1_ × X_5_ + 4.61 × 10^−2^ × X_2_ × X_3_ − 0.184 × X_2_ × X_4_ + 2.57 × 10^−2^ × X_2_ × X_5_ + 0.196 × X_3_ × X_4_ + 7.24 × 10^−2^ × X_3_ × X_5_ − 9.25 × 10^−2^ × X_4_ × X_5_
regression equation of creep recovery rate	Y_5_ = −50.358 − 1.12 × 10^−4^ × X_1_ + 0.160 × X_2_ − 1.083 × X_3_ − 0.878 × X_4_ + 0.748 × X_5_ + 2.8 × 10^−4^ × X_2_^2^ + 2.52 × 10^−2^ × X_3_^2^ − 3.78 × 10^−2^ × X_4_^2^ − 2.43 × 10^−3^ × X_5_^2^ − 8 × 10^−6^ × X_1_ × X_2_ + 6.5 × 10^−5^ × X_1_ × X_3_ + 6.1 × 10^−5^ × X_1_ × X_4_ − 7 × 10^−6^ × X_1_ × X_5_ − 1.72 × 10^−3^ × X_2_ × X_3_ − 2.55 × 10^−4^ × X_2_ × X_4_ − 8.69 × 10^−4^ × X_2_ × X_5_ + 4.20 × 10^−2^ × X_3_ × X_4_ + 3.57 × 10^−3^ × X_3_ × X_5_ + 5.04 × 10^−3^ × X_4_ × X_5_	Y_5_ = −146.464 − 4.88 × 10^−3^ × X_1_ − 1.68 × 10^−2^ × X_2_ − 0.597 × X_3_ + 0.508 × X_4_ + 2.342 × X_5_ + 1 × 10^−6^ × X_1_^2^ + 3.9 × 10^−5^ × X_2_^2^ + 4.6 × 10^−2^ × X_3_^2^ − 2.84 × 10^−2^ × X_4_^2^ − 8.52 × 10^−3^ × X_5_^2^	Y_5_ = −19.031 − 3.4 × 10^−5^ × X_1_ + 0.221 × X_2_ − 0.521 × X_3_ − 1.787 × X_4_ + 0.151 × X_5_ − 1.2 × 10^−5^ × X_1_ × X_2_ + 9.8 × 10^−5^ × X_1_ × X_3_ + 1.15 × 10^−4^ × X_1_ × X_4_ − 1 × 10^−6^ × X_1_ × X_5_ − 2.75 × 10^−3^ × X_2_ × X_3_ − 7.99 × 10^−4^ × X_2_ × X_4_ − 1.06 × 10^−3^ × X_2_ × X_5_ + 5.01 × 10^−2^ × X_3_ × X_4_ + 3.51 × 10^−3^ × X_3_ × X_5_ + 7.24 × 10^−3^ × X_4_ × X_5_

**Table 8:** Optimization solution and corresponding dependent variable in each regression model.

Regression Model and Calculation Method		Partial Least Square Quadratic Term Regression Model	Partial Least Square Interaction Term Regression Model	
		B-1	B-2	B-3
optimization solution	shear rate X_1_/r.p.m.	6500	7000	6500
	shear time X_2_/min	180	200	30
	graphene mixing amount X_3_/‰	20	20	20
	stearic amide mixing amount X_4_/%	1.00	8.26	10.00
	shear temperature X_5_/°C	140	160	150
value of dependent variable	penetration index Y_1_/0.1 mm	88.15	51.27	66.22
	fracture energy Y_2_/N·mm	4301.6	3927.4	3541.9
	softening point Y_3_/	47.51	52.68	51.39
	64 °C antirutting factor Y_4_/kPa	2099.27	2338.77	1909.48
	0.1 kPa creep recovery rate Y_5_/%	30.13	9.25	23.43

**Table 9:** Performance test results of optimal formula.

Item		SK70# Matrix Asphalt	Partial Least Square Quadratic Term Regression Model		Partial Least Square Interaction Term Regression Model			
			B-1	Change Rate/%	B-2	Change Rate/%	B-3	Change Rate/%
penetration/0.1 mm		64.7	61.5	−4.95	62.3	−3.71	58.6	−9.43
softening point/°C		48.1	58.6	21.83	52.3	8.73	54.3	12.89
5 °C force ductility	maximum force/N	96.6	168.0	73.91	136.0	40.79	123.0	27.33
	ductility/mm	6.11	42.54	596.24	44.21	623.57	48.39	691.98
	fracture energy/N·mm	387.7	4035.7	940.93	3542.4	813.70	3358.3	766.21
64 °C antirutting factor/Pa		1442.22	2099	45.54	1643	13.92	1443	0.05
0.1 kPa creep recovery rate/%		2.19	20.24	824.20	8.75	299.54	7.93	262.10
